# Efficient synthesis of limonene production in* Yarrowia lipolytica* by combinatorial engineering strategies

**DOI:** 10.1186/s13068-024-02535-z

**Published:** 2024-07-03

**Authors:** Young-Kyoung Park, Lara Sellés Vidal, David Bell, Jure Zabret, Mladen Soldat, Martin Kavšček, Rodrigo Ledesma-Amaro

**Affiliations:** 1 Department of Bioengineering and Centre for Synthetic Biology, Imperial College London, London, SW72AZ UK; 2 INRAE, AgroParisTech, Micalis Institute, Université Paris-Saclay, 78350 Jouy-en-Josas, France; 3 SynbiCITE Innovation and Knowledge Centre, Imperial College London, London, SW7 2AZ UK; 4 Acies Bio d.o.o., 1000 Tehnološki Park 21Ljubljana, Slovenia

**Keywords:** Limonene, Monoterpene, *Yarrowia lipolytica*, Compartmentalization, Peroxisome, Bioproduction, Synthetic biology, Metabolic engineering, Precision fermentation

## Abstract

**Background:**

Limonene has a variety of applications in the foods, cosmetics, pharmaceuticals, biomaterials, and biofuels industries. In order to meet the growing demand for sustainable production of limonene at industry scale, it is essential to find an alternative production system to traditional plant extraction. A promising and eco-friendly alternative is the use of microbes as cell factories for the synthesis of limonene.

**Results:**

In this study, the oleaginous yeast *Yarrowia lipolytica* has been engineered to produce d- and l-limonene. Four target genes, *l*- or *d*-*LS* (limonene synthase), *HMG* (HMG-CoA reductase), *ERG20* (geranyl diphosphate synthase), and *NDPS1* (neryl diphosphate) were expressed individually or fused together to find the optimal combination for higher limonene production. The strain expressing HMGR and the fusion protein ERG20-LS was the best limonene producer and, therefore, selected for further improvement. By increasing the expression of target genes and optimizing initial OD, 29.4 mg/L of l-limonene and 24.8 mg/L of d-limonene were obtained. We also studied whether peroxisomal compartmentalization of the synthesis pathway was beneficial for limonene production. The introduction of d-LS and ERG20 within the peroxisome improved limonene titers over cytosolic expression. Then, the entire MVA pathway was targeted to the peroxisome to improve precursor supply, which increased d-limonene production to 47.8 mg/L. Finally, through the optimization of fermentation conditions, d-limonene production titer reached 69.3 mg/L.

**Conclusions:**

In this work, *Y. lipolytica* was successfully engineered to produce limonene. Our results showed that higher production of limonene was achieved when the synthesis pathway was targeted to the peroxisome, which indicates that this organelle can favor the bioproduction of terpenes in yeasts. This study opens new avenues for the efficient synthesis of valuable monoterpenes in *Y. lipolytica*.

**Supplementary Information:**

The online version contains supplementary material available at 10.1186/s13068-024-02535-z.

## Background

Limonene is a well-known monoterpene composed of two isoprene (C5) units. Both optical forms of limonene are present in essential oils derived from various plant species. The main application of limonene has been as flavor and fragrance ingredients in cosmetics and foods. The flavor characteristics can vary depending on the chirality and source of limonene [1, 2]. As well as its traditional use as a flavor, limonene has diverse applications in pharmaceuticals as anti-microbial and anti-cancer compounds, and in chemical and food industries as a resin and masticatory agent [2–4]. Furthermore, polymers derived from limonene are utilized in various industries as adhesives, sealants, metal coatings, and printing inks [2]. In addition, limonene serves as a precursor for valuable compounds such as perillyl alcohol, menthol, carveol, and α-terpineol, which have significant applications in the foods, cosmetics, pharmaceuticals, biomaterials, and biofuels industries [5]. Therefore, there is a growing demand for sustainable production of limonene at a large scale to meet these diverse industry needs.

Plants have traditionally been the primary sources of limonene and other terpenes. However, plant-derived production faces several limitations including low yield, dependency on seasonal and climatic conditions, high production costs (including downstream processing), and environmental pollution resulting from complex extraction processes [2, 6]. Chemical synthesis of limonene also suffers from its own drawbacks including high energy consumption or environmental damage [1, 2, 6]. Therefore, the microbial production of limonene by synthetic biology has emerged as a promising alternative in terms of sustainability and economic feasibility. Various strategies have been employed to produce limonene, including overexpressing heterologous or native genes in target pathways, increasing the copy number of limonene synthase genes and improving the tolerance to limonene [4, 6, 7]. However, despite these efforts, achieving a high production of limonene still remains a significant challenge.

The selection of a suitable microbial host plays a crucial role in bioproduction. Factors such as the presence of native precursor pathways, tolerance to intermediate and final compounds, and genetic amenability are important considerations in this selection process. *Yarrowia lipolytica*, a non-conventional yeast, possesses distinctive traits that makes it a good host for industrial bioproduction [8–10]. Due to its safety, robustness, efficient genetic modifications, and broad range of possible substrates, *Y. lipolytica* has strengths as a host microorganism for bioproduction [8, 10]. In addition, high carbon flux toward acetyl coenzyme A (acetyl-CoA) and NADPH and a hydrophobic microenvironment make *Y. lipolytica* an organism of choice for terpene or lipid production [7, 8, 11, 12].

In *Y. lipolytica*, the production of limonene has been achieved by introducing heterologous limonene synthases (LS) from diverse origins. Since the sole expression of LS was insufficient to have the desired levels of production in many cases, further metabolic engineering strategies have been employed. These include overexpressing genes in the mevalonate (MVA) pathway to boost the limonene precursors and expressing the limonene synthase genes at high copy numbers. To improve acetyl-CoA and upregulate the MVA pathway, Arnesen and colleagues overexpressed several native or heterologous genes encoding ACL1 (ATP citrate lyase), ACS (acetyl-CoA synthetase from *Salmonella enterica*), HMG (3-hydroxy-3-methylglutaryl-CoA reductase), ERG12 (mevalonate kinase), IDI (isopentyl diphosphate isomerase), ERG20 (farnesyl diphosphate synthase, mutated), and lowered the expression of SQS (squalene synthase) in *Y. lipolytica* [11]. The expression of LS from *Perilla frutescens* in this platform strain resulted in the production of 35.9 mg/L of limonene. In another study, the carbon flux from isopentenyl diphosphate (IPP) and dimethylallyl diphosphate (DMAPP) was redirected towards neryl diphosphate (NPP) by introducing NPP synthase (NDPS1 from *Solanum lycopersicum*) into *Y. lipolytica* [12]. Through a combination of strain engineering, involving the overexpression of d-LS, HMG1, and ERG12, and media optimization including testing different carbon sources and using a dodecane overlay, the limonene production reached 23.56 mg/L. The same group further developed the strain by expressing two copies of d-LS and optimizing the fermentation condition resulting in an increase in limonene up to 165.3 mg/L [13]. In efforts to enhance the cost-effectiveness of limonene production, low-cost substrates have been utilized in combination with metabolic engineering strategies in *Y. lipolytica*. In these studies, limonene production levels of 20.57 mg/L and 91.24 mg/L were produced from lignocellulosic hydrolysates and waste cooking oils, respectively [14, 15].

In this study, we present two distinct strategies for enhancing limonene production in *Y. lipolytica*. The first strategy involves the fusion of limonene synthase and ERG20m to improve limonene production. The multicassette overexpression of MVA pathway genes coupled with the fusion enzyme was shown as an effective strategy in increasing limonene production. The second strategy focuses on compartmentalizing limonene production within the peroxisome. This approach aims to minimize competition between native metabolic pathways and limonene synthesis. By implementing the MVA pathway in the peroxisome along with LS, a significant increase in limonene production was shown. In addition, we have optimized the culture condition for our best-performing strain to maximize production.

## Results

### Selection of MVA gene to boost limonene production

**Fig. 1:**
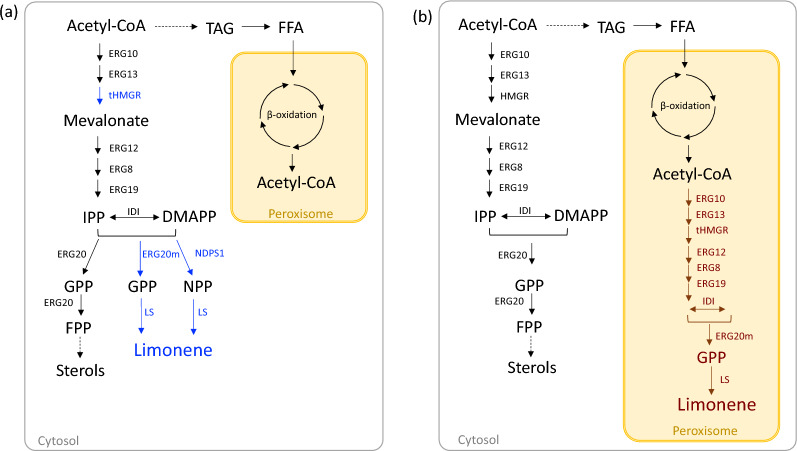
Synthetic pathway of limonene with two different strategies. **a** Limonene production through investigation of the genes in native mevalonate pathway or heterologous genes was carried out in cytosol (in grey). **b** The strategy of limonene production by establishing the mevalonate pathway and limonene synthase in the peroxisome (in yellow). The overexpressed genes are in blue (cytosolic expression) or brown (peroxisomal expression). *TAG* triacylglycerol, *FFA* free fatty acid, *IPP* isopentenyl diphosphate, *DMAPP* dimethylallyl diphosphate, *GPP* geranyl diphosphate, *NPP* neryl diphosphate, *FPP* farnesyl diphosphate, *ERG10* acetyl-CoA acetyltransferase, *ERG13* HMG-CoA synthase, *tHMGR* truncated HMG-CoA reductase, *ERG12* mevalonate kinase, *ERG8* phosphomevalonate kinase, *ERG19* mevalonate diphosphate decarboxylase, *IDI* isopentenyl diphosphate delta-isomerase, *ERG20m* geranyl diphosphate synthase with mutation, *LS* limonene synthase, *NDPS1* neryl diphosphate synthase

In this study, we expressed two limonene synthases (d-form from *Citrus limon*, l-form from *Mentha spicata*) in *Y. lipolytica* after codon-optimization. Previous studies have demonstrated that the production of monoterpene often requires the expression of genes in the MVA pathway, as well as the heterologous limonene synthase gene, to provide the necessary precursor pools (Fig. 1a). Thus, we specifically targeted three genes, *HMGR*, *ERG20*, and *NDPS1* to improve the limonene synthesis. HMGRp (3-hydroxy-3-methylglutaryl-Coenzyme A reductase) has been identified as a key enzyme for terpene synthesis [7, 15]. In previous studies, the truncation of the N-terminal domain of HMGRp leads to increased terpene production by providing better soluble expression [16]. In our study, we utilized the truncated version of HMPRp (tHMGRp) to assess its impact on limonene production. ERG20p (geranyl diphosphate synthase) is a bifunctional enzyme responsible for two consecutive reactions that form GPP and FPP. Overexpression of *ERG20* often resulted in a moderate increase on monoterpene production because the GPP pool is insufficient. Mutations in ERG20p (*ERG20*^*F88W−N119W*^, ERG20m in this study) have been found to enhance GPP availability resulting in higher levels of monoterpenes in *S. cerevisiae* and *Y. lipolytica* [11, 12, 17, 18]. In addition, we considered employing an alternative pathway, known as the orthogonal pathway, to bypass the native sterol pathway to improve the limonene synthesis. This approach involved the overexpression of *NDPS1* from *Solanum lycopersicum* to synthesize neryl diphosphate (NPP), an alternative precursor [19]. The biosynthesis of monoterpene derived from NPP has demonstrated an increase in monoterpene production in *S. cerevisiae* and *Y. lipolytica* [12, 19]. Furthermore, enzyme fusion has been investigated as a strategy to facilitate the conversion of precursors into terpenes [1, 6]. Thus, we expressed two fusion enzymes, ERG20m/(d)-LS or ERG20m/(l)-LS which fused ERG20m to N-term of limonene synthase with linker ‘GSGSGSGSGS’, to evaluate their potential for improving limonene production.

**Fig. 2:**
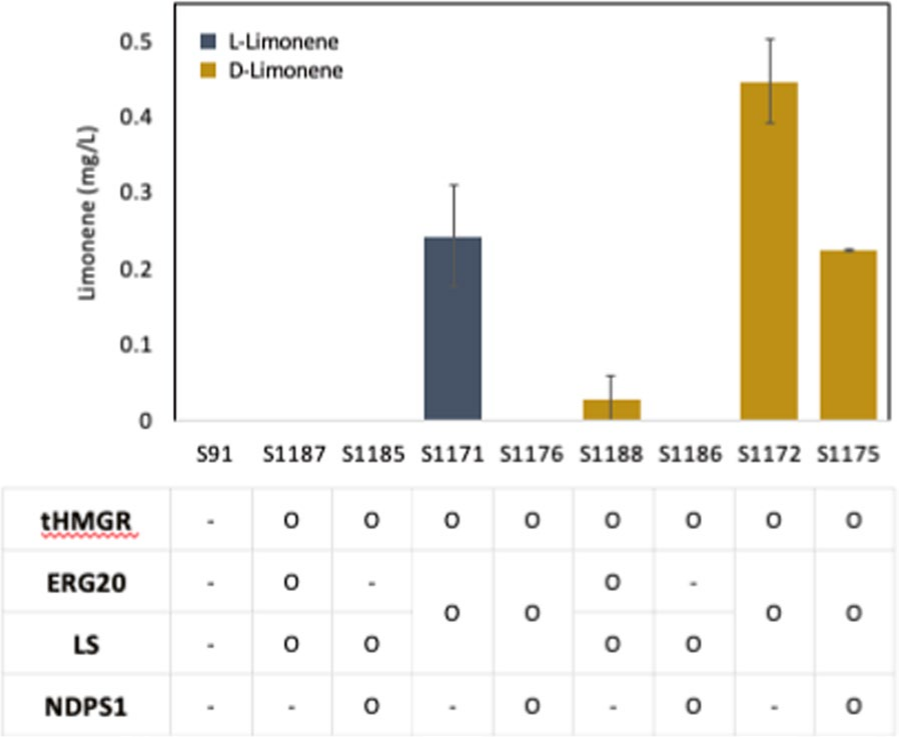
The effects of overexpressing the native or heterologous genes involved in the MVA pathway on l- and d-limonene production. l-limonene is in navy and d-limonene is in dark yellow. The strains were cultivated in a YPG (2% glycerol) medium for 5 days. The values show the average and standard deviation of two biological replicates

In the case of l-limonene, we observed that limonene production was only detected in the strain harboring the fusion enzyme ERG20m/(l)-LS and tHMGRp, as shown in Fig. 2. However, the combination of enzymes such as tHMGR, ERG20m with (l)-LS, or tHMGR, NDPS1 with (l)-LS did not result in limonene production. Regarding d-limonene, the overexpression of *tHMGR*, *ERG20m*, and *(d)-LS* led to a very low level of d-limonene. However, when NDPS1 was overexpressed instead of ERG20m, d-limonene production was not observed. Interestingly, the fusion of ERG20m and (d)-LS (S1172) exhibited a 14.8-fold increase in limonene production compared to individual overexpression (S1188). Further overexpression of *NDPS1* (S1175) did not result in an increase in limonene production. The introduction of the orthogonal pathway by overexpressing *NDPS1* commonly had a negative effect on the production of both l- and d-limonene in this study.

### Increase the expression level

**Fig. 3:**
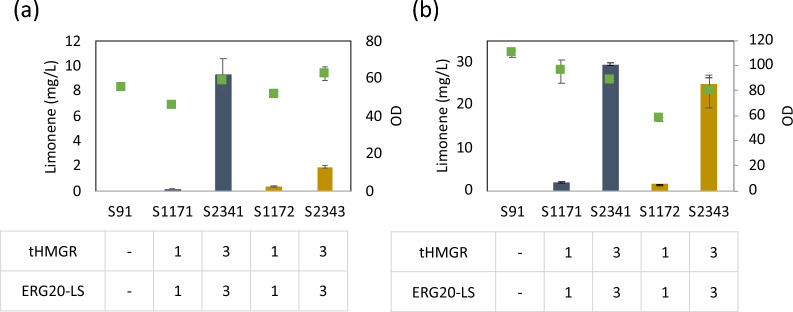
Effects of expression cassette number on l- and d-limonene production. **a** Limonene production depending on different expression cassette numbers. The strains were cultivated in a YPG (2% glycerol) medium for 5 days with the initial OD 0.1. **b** Limonene production depending on different numbers. The strains were cultivated in a YPD (4% glucose) medium for 5 days with the initial OD 1.0. l-Limonene, navy bar; d-limonene, dark yellow bar; and OD, green dot. The values show the average and the standard deviation of the two biological replicates

To enhance the production of limonene, we conducted multiround integration of the best producing cassette, tHMGR and the fusion enzyme of ERG20m and LS, as illustrated in Fig. 3a. Three expression cassettes of selected genes with different selective markers were randomly integrated in the genome of *Y. lipolytica*. By increasing the number of transformation events with the expression cassettes with different selective markers, we observed a significant increase in both forms of limonene (l- and d-) by 51.8- and 5.3-fold, respectively (Fig 3b). To further investigate the potential for increased production and to demonstrate the effect more clearly, we cultivated the strains under conditions with a high glucose concentration (4%) and a high initial OD of 1.0. Under this condition, multi-cassette integration led to a 15.2- and 16.0-fold increase in l- and d-limonene, respectively (Fig 3b). The highest production reached 29.4 mg/L in l-limonene from the S2341 strain and 24.8 mg/L of d-limonene from the S2343 strain. These findings highlight the effectiveness of increasing expression level in enhancing the production of both l- and d-limonene.

### Compartmentalization of limonene synthesis

Peroxisome has been identified as a promising organelle for terpene production [20]. This is primarily attributed to its high abundance of acetyl-CoA derived from β-oxidation and its ability to sequester toxic molecules, thereby detoxifying the rest of the cell [21–23].

**Fig. 4:**
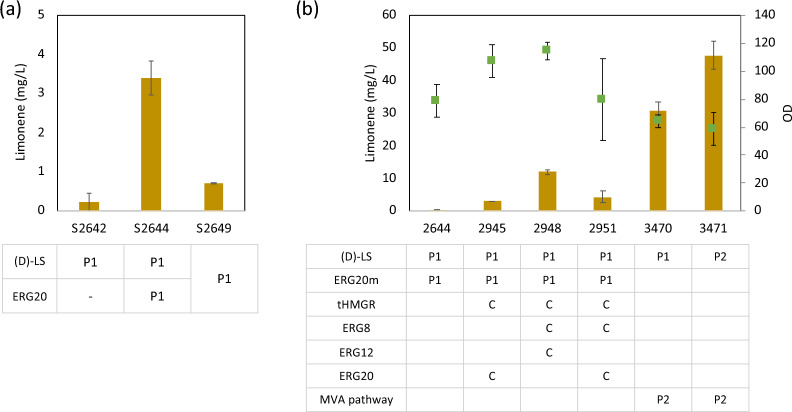
Limonene production by targeting gene expression in peroxisome. **a** The effect of overexpressing *d-LS* and *ERG20m* in peroxisome in d-limonene production. **b** The effect of overexpressing *d-LS* in peroxisome with overexpressing genes in mevalonate pathway in cytosol or peroxisome. l-limonene, navy bar; d-limonene, dark yellow bar; OD, green dot; P1, peroxisomal expression with PTS1; P2, peroxisomal expression with PTS2; C, cytosolic expression. The strains were cultivated in a YPD (4% glucose) medium for 5 days. The values show the average and the standard deviation of the two biological replicates

To localize the expression of two genes, D-LS and ERG20m, in the peroxisome of *Y. lipolytica*, a peroxisomal targeting sequence (PTS1, MGAGVTEDQFKSKL from *ICL1*) was added [24]. Individual expressions of Dd-LS and ERG20m in the peroxisome (S2644) yielded the highest limonene production at 3.4 mg/L without changes in cell growth (Fig. 4a). This co-expression resulted in a 14.9-fold increase in limonene production compared to the single overexpression of d-LS (S2642). Notably, the expression of fusion enzyme ERG20m/d-LS in peroxisome (S2649) led to a 4.8-fold increase in limonene production compared to the single overexpression of d-LS (S2642), but the level of limonene was lower than those observed in the individual expression of ERG20m and d-LS (S2644).

The single-layer membrane of peroxisome allows for the passage of low molecular weight compounds, facilitating the utilization of intermediates from cytosol for limonene production. The overexpression of key enzymes (d-LS and ERG20m) in peroxisome enabled the production of limonene by utilizing intermediates from cytosol. To test the availability of a high pool of intermediates in the MVA pathway, several combinations of genes in the MVA pathway were overexpressed in the cytosol along with *d-LS* and *ERG20m* in the peroxisome. The production of limonene was increased from 0.5 mg/L to 3.0, 4.2, and 11.9 mg/L by overexpressing *tHMG* + *ERG20*, *tHMG* + *ERG8* + *ERG12*, and *tHMG* + *ERG8* + *ERG20*, respectively. Among the combinations tested in this study, the overexpression of *tHMGR*, *ERG8*, and *ERG12* in the cytosol (S2948) resulted in the highest increase in limonene production, reaching a level 39.7 times higher than that without the boosting of the cytosolic MVA pathway (S2644). The results suggest that the precursors of limonene can be translocated from cytosol to peroxisome in *Y. lipolytica* as previously observed in *S. cerevisiae* [21].

To investigate whether a direct precursor supply in the peroxisome can lead to further improved limonene synthesis, the entire MVA pathway was overexpressed in the peroxisome using two different PTSs. PTS2 (GGGSSKL) was utilized to localize the entire MVA pathway in the peroxisome [22]. In addition, both PTS1 and PTS2 were employed for the expression of D-LS to determine which PTS is more effective for limonene production. The expression of the entire MVA pathway in the peroxisome resulted in a significant increase in limonene production compared to co-expression of *d-LS* and *ERG20m* in peroxisome (S2644). There was a 102.6-fold increase in limonene production from d-LS_PTS1_ and a 159.3-fold from d-LS_PTS2_ compared to the control. The highest limonene titer reached 47.8 mg/L in the strain S3471, which is 8.1 times higher than the best-performing strain achieved by a multiround integration in the cytosol (S2343) under this experimental condition.

### Fed-batch fermentation

**Fig. 5:**
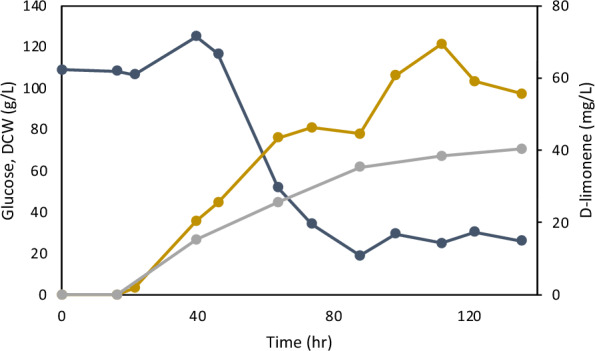
Time course of d-limonene production of the engineered *Y. lipolytica* strain in fed-batch fermentation. The strain was cultivated at 28 °C, pH 5.4 with an initial OD 6. Glucose level was maintained at around 20 g/L through feeding. Glucose, navy; d-Limonene, dark yellow; DCW, grey

We evaluated the D-limonene production of the best-performing strain (S3471) harboring the peroxisomal pathway in fed-batch cultivation. The fed-batch fermentations were performed using a YP media with an initial glucose concentration of 100 g/L. Glucose was fed to maintain the level around 20 g/L. The fed-batch fermentation resulted in a continuous accumulation of d-limonene that was proportional to the biomass (Fig. 5). The highest titer of d-limonene, 69.3 mg/L, was achieved at 120 h of cultivation which represents the production of d-limonene at 1.81 mg/g DCW. The result demonstrated that peroxisomal engineering and fed-batch cultivation are promising strategies for limonene production in *Y. lipolytica*.

## Discussion

Limonene boasts a substantial industrial exploitation value, finding applications in the fragrance, pharmaceutical, and food industries [1, 25, 26]. While traditionally sourced from plants, there is a growing interest in microbial production circumventing the drawbacks associated with plant-derived extraction. However, the microbial synthesis of limonene has posed challenges, primarily coming from the low expression of the heterologous enzyme, competition with native metabolic pathways, the inherent toxicity of limonene to host cells, and so on [1]. Previous research endeavors into limonene biosynthesis have explored diverse strategies, including enhancing the MVA pathway, increasing acetyl-CoA availability, and mitigating the toxicity of limonene. Here, we explored the biosynthesis of limonene within cytosol or the peroxisome of *Y. lipolytica* as a promising strategy to address these challenges.

For cytosolic production of limonene, we elevated the expression of key enzymes, namely LS, tHMGR, and ERG20m. The sole expression of LS did not yield detectable limonene which is consistent with prior studies. However, limonene was detected upon co-expression of a fusion protein comprised of ERG20m and LS, which was not the case in the separate expression of these two enzymes. It is noteworthy that protein fusion, a strategy often employed for enzymes catalyzing sequential reactions, serves to enhance substrate channeling, minimize intermediates loss, and thus improve overall enzyme activity. This approach has previously had demonstrated success in the production of various terpenes including farnesene, geraniol, and sabinene [16, 18, 27]. Particularly in the context of monoterpene synthesis, the limited availability of GPP, a pivotal precursor for monoterpene production, has been identified as a bottleneck due to the bifunctional enzyme ERG20p. This enzyme’s proclivity for diverting GPP towards the formation of FPP rather than monoterpene compounds can lead to inefficiencies in monoterpene synthesis. The fusion of ERG20p and LSp, as applied in this study, offers a solution by promptly sequestering GPP and directing its conversion to limonene before it can be used in FPP synthesis. Furthermore, the significant enhancement of limonene production, by 5.3- and 51.8-fold, was achieved through the elevated expression of target genes by multiple integration. This strategy represents a synergistic approach that combines the strengthening of the upstream pathway, fusion protein-mediated precursor supply, and the enhancement of expression of the key enzymes. These results align with previous studies that have improved the production of target compounds [14, 22, 28].

Utilizing organelle engineering to compartmentalize partial or complete biosynthetic pathways presents distinct advantages when compared to rewiring cytoplasmic metabolic pathways. This approach offers a conducive physicochemical environment for target compound synthesis, ensuring an adequate supply of precursors or enzymes [22, 29]. Peroxisomes, in particular, emerge as a valuable organelle for terpene production, owing to their rich reserves of acetyl-CoA, a critical precursor for terpene biosynthesis. In addition, the separation of monoterpene synthesis from GPP within peroxisome, distinct from the native pathway employing GPP in cytosol, effectively minimizes competition [21]. In this study, we introduced LS into the peroxisome both with and without the inclusion of MVA pathway enzyme. The overexpression of D-LS and ERG20m within peroxisome led to an enhancement in limonene production, yielding 3.4 mg/L. This represents a significant improvement over strains expressing the target genes in the cytosol (Fig. 2), underscoring the efficacy of peroxisome-based engineering for monoterpene synthesis. Additional overexpression of MVA pathway genes in the cytosol also showed an increase in the limonene titer, ranging from 10- to 39.5-fold, a finding consistent with the previous study carried out in *S. cerevisiae* [21]. This suggests that intermediates from the MVA and terpene biosynthetic pathways can be effectively transported into the peroxisome in *Y. lipolytica*. To further enhance precursor transport, the implementation of engineering strategies, such as channeling proteins, including peroxisomal ATP-binding cassette transporters (PXA1 and PXA2), can be considered to increase production further [30]. We observed a substantial increase in limonene production through the incorporation of the entire MVA pathway within the peroxisome, achieving 47.8 mg/L. This shows the potential of peroxisomes as a key organelle for monoterpene production in *Y. lipolytica*. Future strategies may include peroxisome engineering to increase its size and quantity [29] and the optimization of cofactor supply (ATP, NADPH) [22, 26, 30].

Monoterpenes, including limonene, often exhibit toxicity to cells by affecting membrane integrity, a phenomenon recognized as a significant impediment to achieve high-titer production [1, 3]. In this study, we applied a two-phase cultivation approach, incorporating dodecane to mitigate the toxic effect of the produced limonene on the cell. However, even with the dodecane phase, we observed a reduction in biomass in the strain containing the MVA pathway within the peroxisome. This might be attributed to an internal metabolic imbalance and external environment interference, potentially stemming from metabolic burden [25]. This result contrasts with previous result where growth was maintained after the implantation of the entire MVA pathway within peroxisome for producing sesquiterpene, α-humulene, in *Y. lipolytica* [22]. In other contexts, engineering peroxisomes as a production module for fatty alcohol in *Ogataea polymorpha* resulted in reduced growth [30]. However, the introduction of further engineering strategies aimed at reducing stress on peroxisome homeostasis, enhancing precursor and cofactor supply, led to improved growth and production. Consequently, further peroxisome engineering holds promise for alleviating limonene toxicity and improving biomass and production.

To further enhance limonene production, it would be interesting to explore several strategies aimed at increasing acetyl-CoA levels, a tactic previously demonstrated to be effective for improving the production of farnesene and squalene [31–34]. Moreover, it is important to consider that monoterpene synthesis necessitates four molecules of NADPH, six molecules of ATP, and six molecules of acetyl-CoA. Therefore, engineering cofactor availability by modifying the pentose phosphate pathway or inhibiting NADPH-consuming pathway may contribute to more efficient and productive limonene biosynthesis, as shown in *S. cerevisiae* [25, 26].

## Conclusions

Limonene is of great interest in the field of biotechnology due to its versatile application. However, achieving microbial production of limonene at economically feasible titers remains a substantial challenging. Here, *Y. lipolytica* was engineered to produce limonene by metabolic engineering both in the cytosol or peroxisome. By combining precursor supply enhancements with elevated gene expression, we accomplished the biosynthesis of d- and l-limonene, yielding 24.8 mg/L and 29.4 mg/L, respectively, in flask cultivation. Notably, the strategic incorporation of peroxisomal compartmentalization elevated d-limonene production, reaching 47.8 mg/L in flask cultivation. Through the fed-batch fermentation, we achieved a yield of 69.3 mg/L of d-limonene. This study presents a pioneering approach of using peroxisomes as a platform for limonene production in *Y. lipolytica* and opens new avenues for the efficient synthesis of other monoterpenes in *Y. lipolytica* via harnessing the high potential of organelle compartmentalization strategies.

## Materials and methods

### Strains, media, and culture conditions

The *E. coli* strain DH5α and TOP10 were used as the host in this study for the cloning and plasmid construction. *E. coli* strains were grown at 37 °C in Luria–Bertani (LB) medium (containing 1% tryptone, 0.5% yeast extract, and 1% sodium chloride) or on an LB agar plate. When necessary, appropriate antibiotics such as chloramphenicol, spectinomycin, ampicillin, or kanamycin were added at concentrations of 34 µg/mL, 50 µg/mL, 100 µg/mL, and 50 µg/mL, respectively.

*Y. lipolytica* was routinely grown at 30 °C in YPD medium which consists of 1% yeast extract, 2% peptone, and 2% glucose, or yeast synthetic medium (YNBD) which includes 0.17% yeast nitrogen base without amino acids and ammonium sulfate, 0.5% ammonium chloride, 50 mM phosphate buffer (KH_2_PO_4_–Na_2_HPO_4_, pH 6.8), and 2% glucose. To prepare the solid medium, 1.5% agar was added to the respective liquid medium. To complement auxotrophic processes, uracil, leucine, or tryptophan was added at a concentration of 0.1 g/L, as necessary. The strains and plasmids used in this study are listed in Table 1.

**Table 1:** Plasmids and strains used in this study

Plasmid	Description	References
RLA P995	ZUS1.2-pTEF-ERG20-TLip2	This study
RLA P996	ZUS1.2-pTEF-ERG20/(D)LS-TLip2	This study
RLA P997	ZUS1.2-pTEF-ERG20/(L)LS-TLip2	This study
RLA P998	ZUS1.2-pTEF-NDPI-TLip2	This study
RLA P999	ZUS1.2-pTEF-(D)LS-TLip2	This study
RLA P1000	ZLA2.II-pTEF-tHMG-TLip2-pTEF-ERG20/(L)LS-TLip2	This study
RLA P1001	ZLA2.II-pTEF-tHMG-TLip2-pTEF-ERG20/(D)LS-TLip2	This study
RLA P1022	ZUS1.1-pTEF-tHMG-TLip2	This study
RLA P1063	ZUS1.3-pTEF-ERG20/(D)LS-TLip2	This study
RLA P1064	ZUS1.3-pTEF-ERG20/(L)LS-TLip2	This study
RLA P1065	ZUA2.III pTEF-tHMG-TLip2-pTEF-NDPI-TLip2-pTEF-Erg20/(L)LS-TLip2	This study
RLA P1226	pZLA2.III-pTEF-tHMG-TLip2-pTEF-ERG8-TLip2-pTEF-ERG19-TLip2	This study
RLA P1440	pZLA2.II-pTEF-tHMG-TLip2-pTEF-ERG20-TLip2	This study
RLA P1441	ZLA2.III-pTEF-tHMG-TLip2-pTEF-ERG20-TLip2-pTEF-(L)LS-TLip2	This study
RLA P1442	ZLA2.III-pTEF-tHMG-TLip2-pTEF-NDPI-TLip2-pTEF-(L)LS-TLip2	This study
RLA P1534	ZLA2.III-pTEF-tHMG-TLip2-pTEF-ERG8-TLip2-pTEF-ERG12-TLip2	This study
RLA P2224	ZUS1.1-pTEF-ERG20pero-TLip2	This study
RLA P2225	ZUS1.2-pTEF-(D)LSpero-TLip2	This study
RLA P2229	ZLS1.1-pTEF-ERG20pero-TLip2	This study
RLA P2232	ZUS1.2-pTEF-ERG20/(D)LSpero-TLip2	This study
RLA P2693	ZUA2.III-pTEF-IDI-TLip2-pTEF-ERG20-TLip2-pTEF-ERG19-TLip2	This study
RLA P2695	ZTA2.III-pTEF-ERG10-TLip2-pTEF-(D)LS-TLip2-pTEF-ERG13-TLip2	This study
RLA P2697	ZT4A2.III-pTEF-ERG10-TLip2-pTEF-(D)LSpero-TLip2-pTEF-ERG13-TLip2	This study
RLA P2698	ZT4A2.III-pTEF-ERG10-TLip2-pTEF-(D)LSpero2-TLip2-pTEF-ERG13-TLip2	This study
RLA P2749	ZLA2.III-pTEF-tHMGpero-TLip2-pTEF-ERG8pero-TLip2-pTEF-ERG12pero-TLip2	This study
RLA P2750	ZUA2.III-pTEF-IDIpero-TLip2-pTEF-ERG20pero-TLip2-pTEF-ERG19pero-TLip2	This study
RLA P2752	ZTA2.III-pTEF-ERG10pero-TLip2-pTEF-(D)LSpero-TLip2-pTEF-ERG13pero-TLip2	This study
RLA P2753	ZTA2.III-pTEF-ERG10pero-TLip2-pTEF-(D)LSpero2-TLip2-pTEF-ERG13pero-TLip2	This study

*Y. lipolytica*	Description	This study
RLA S3	Po1d MATA ura3-302 leu2-270 xpr2-322	This study
RLA S1708	Po1d MATA ura3-302 leu2-270 xpr2-322 Δtrp4	This study
RLA S1171	Po1d ZLA-pTEF-tHMG-TLip2-pTEF-Erg20/(L)LS-TLip2 + URA3	This study
RLA S1172	Po1d ZLA-pTEF-tHMG-TLip2-pTEF-Erg20/(D)LS-TLip2 + URA3	This study
RLA S1173	Po1d ZLA-pTEF-tHMG-TLip2-pTEF-NDPI-TLip2-pTEF-(D)LS-TLip2 + URA3	This study
RLA S1175	Po1dZUA-pTEF-tHMG-TLip2-pTEF-NDPI-TLip2-pTEF-Erg20/(D)LS-TLip2 + LEU2	This study
RLA S1176	Po1d ZUA-pTEF-tHMG-TLip2-pTEF-NDPI-TLip2-pTEF-Erg20/(L)LS-TLip2 + LEU2	This study
RLA S1185	po1d + ZLA2.III-pTEF-tHMG-TLip2-pTEF-NDPI-TLip2-pTEF-(L)LS-TLip2 + URA3	This study
RLA S1186	po1d + ZLA2.III-pTEF-tHMG-TLip2-pTEF-NDPI-TLip2-pTEF-(D)LS-TLip2 + URA3	This study
RLA S1187	po1d + ZLA2.III-pTEF-tHMG-TLip2-pTEF-ERG20-TLip2-pTEF-(L)LS-TLip2 + URA3	This study
RLA S1188	po1d + ZLA2.III-pTEF-tHMG-TLip2-pTEF-ERG20-TLip2-pTEF-(D)LS-TLip2 + URA3	This study
RLA S2285	Po1d pTEF-tHMG-TLip2-pTEF-Erg20/(L)LS-TLip2	This study
RLA S2286	Po1d pTEF-tHMG-TLip2-pTEF-Erg20/(D)LS-TLip2	This study
RLA S2341	Po1d (pTEF-tHMG-TLip2-pTEF-Erg20/(L)LS-TLip2)X3cassettes	This study
RLA S2343	Po1d (pTEF-tHMG-TLip2-pTEF-Erg20/(D)LS-TLip2)X3cassettes	This study
RLA S2642	Po1d ZUS1.2-pTEF-(D)LSpero-Tlip2 + LEU2	This study
RLA S2644	Po1d ZUS1.2-pTEF-(D)LSpero-TLip2 ZLS1.1-pTEF-ERG20pero-TLip2	This study
RLA S2649	Po1d ZUS1.2-pTEF-(D)LSpero-Tlip2 + LEU2	This study
RLA S2945	Po1d ZUA2.II-pTEF-(D)LSpero-TLip2-pTEF-ERG20pero-TLip2 + ZLA2.II-pTEF-tHMG-TLip2-pTEF-ERG20-TLip2	This study
RLA S2948	Po1d ZUA2.II-pTEF-(D)LSpero-TLip2-pTEF-ERG20pero-TLip2 + ZLA2.II-pTEF-tHMG-TLip2-pTEF-ERG8-TLip2-pTEF-ERG12-TLip2	This study
RLA S2951	Po1d ZUA2.II-pTEF-(D)LSpero-TLip2-pTEF-ERG20pero-TLip2 + ZLA2.II-pTEF-tHMG-TLip2-pTEF-ERG8-TLip2-pTEF-ERG20-TLip2	This study
RLA S3448	Po1d ZUA2.III-pTEF-IDI-TLip2-pTEF-ERG20-TLip2-pTEF-ERG12-TLip2 + ZLA2.III-pTEF-tHMG-TLip2-pTEF-ERG8-TLip2-pTEF-ERG12-TLip2 ZTA2.III-pTEF-ERG10-TLip2-pTEF-(D)LS-TLip2-pTEF-ERG13-TLip2	This study
RLA S3450	Po1d ZLA2.III-pTEF-tHMG-TLip2-pTEF-ERG8-TLip2-pTEF-ERG12-TLip2 + ZUA2.III-pTEF-IDI-TLip2-pTEF-ERG20-TLip2-pTEF-ERG19-TLip2 ZTA2.III-pTEF-ERG10-TLip2-pTEF-(D)LSpero2-TLip2-pTEF-ERG13-TLip2	This study
RLA S3470	Po1d ZLA2.III-pTEF-tHMGpero-TLip2-pTEF-ERG8pero-TLip2-pTEF-ERG12pero-TLip2 + ZUA2.III-pTEF-IDIpero-TLip2-pTEF-ERG20pero-TLip2-pTEF-ERG19pero-TLip2 ZTA2.III-pTEF-ERG10pero-TLip2-pTEF-(D)LSpero-TLip2-pTEF-ERG13pero-TLip2	This study
RLA S3471	Po1d ZLA2.III-pTEF-tHMGpero-TLip2-pTEF-ERG8pero-TLip2-pTEF-ERG12pero-TLip2 + ZUA2.III-pTEF-IDIpero-TLip2-pTEF-ERG20pero-TLip2-pTEF-ERG19pero-TLip2 ZTA2.III-pTEF-ERG10pero-TLip2-pTEF-(D)LSpero2-TLip2-pTEF-ERG13pero-TLip2	This study

### Construction of plasmids

Restriction enzymes were obtained from New England Biolabs (Ipswich, MA, USA). PCR amplifications were performed in a PCR ProFlex™ (Applied Biosystems, Waltham, USA) with GoTaq DNA polymerases (Promega, Madison, USA) and Q5 High-Fidelity DNA Polymerase (New England Biolabs, Ipswich, USA). PCR fragments were purified with a QIAgen Purification Kit (Qiagen, Hilden, Germany).

The heterologous genes, (d)-Limonene synthase (*Citrus limon*, Sequence ID: AAM53946.1), (l)-LS (*Mentha spicata*, Sequence ID: AAC37366.1), and NDPS1 (*Solanum lycopersicum*, Sequence ID: 7VPC_A) were codon optimized to *Y. lipolytica* and then synthesized by TWIST Biosciences HQ (CA, USA). Native genes were amplified from *Y. lipolytica* by PCR. The sequences of heterologous proteins are listed in the supplementary Table 1.

The plasmids used in this study were constructed by Golden Gate Assembly, as described in Yuzbashev et al. [35]. In brief, each gene was cloned to Lv0 plasmid using BsmBI. Lv1 plasmid containing the specific overhang for Lv2 plasmid was then constructed by assembling the Lv0 plasmid of promoter, gene, and terminator using BsaI. Finally, the Lv2 plasmid containing two or three transcription units was constructed using BsmBI. To verify the correct construction of plasmids, colony PCR and digestion by restriction enzyme were carried out. The primers used for cloning and verification are listed in supplementary Table 2.

### Construction of *Y. lipolytica* strains

To introduce gene expression cassette into *Y. lipolytica*, the plasmids were first linearized using NotI and then transformed into competent cells using the lithium acetate/DTT method. The gene expression cassettes were randomly integrated into the genome of *Y. lipolytica* with the zeta sequence. Transformants were selected on YNBD media containing the appropriate amino acids for their specific genotype. Positive transformants were then confirmed by colony PCR with Phire Plant Direct PCR master mix (Thermo Fisher, Waltham, USA). The removal of the selection marker was carried out via the Cre-LoxP system.

### Cultivation of *Y. lipolytica* for producing limonene at flask scale

*Y. lipolytica* seed cultures were cultivated overnight at 28 °C and 220 rpm in 50 mL culture tubes containing 5 mL of YNBD media, supplemented with the appropriate amino acids if necessary. Pre-cultured cells were inoculated with initial OD at 0.05 in 50 mL of YP medium consisting of 10 g/L yeast extract and 20 g/L peptone with either glucose (40 g/L) or glycerol (20 g/L) as substrate and cultivated at 28 °C and 220 rpm. An overlay of 20% (v/v) dodecane was added to each flask, and the flasks were covered with aluminum foil and sealed with parafilm to prevent the evaporation. We used two biological replicates and calculated the value of average and standard deviation.

### Cultivation of *Y. lipolytica* for producing limonene at a bioreactor scale

The strain was initially cultivated in YNBD medium at 28 °C and 220 rpm overnight. Subsequently, the culture was inoculated into 2000 mL YPD medium (10% glucose) within a 6.6 L Sartorius BIOSTAT bioreactor (Sartorius, Germany), incorporating a 20% (v/v) dodecane phase as an organic extractant. Fermentation conditions were maintained at 28 °C, with agitation speeds ranging from 300 to 900 rpm and an airflow of 2 Lpm, while pH was adjusted to 5.4 using 20% (w/v) KOH or 20% (w/v) H_3_PO_4_. A fed-batch strategy was implemented, maintaining glucose at around 20 g/L by feeding of 70% (w/v) glucose.

### Analysis (OD, Limonene)

Cell growth was monitored by measuring OD at 600 nm using either a spectrophotometer Biowave II (WPA, UK) or a 96-well TECAN Infinite® 200 PRO plate reader (TECAN, CH).

Limonene was quantified by an Accela 1250 pump (Thermo Fischer Scientific, USA) connected to an Accucore C18 column (Thermo Fischer Scientific, USA), heated to 60 °C, and coupled with a TSQ Quantum Access MAX MS/MS mass spectrometer (Thermo Fischer Scientific, USA). The sample injection volume was 10 μL with the mobile phase consisting of 85% (v/v) methanol and 12% (v/v) Milli-Q water with a flow rate of 500 mL/min. Milli-Q water was obtained through a Milli-Q Millipore filter system (Millipore Co., USA). APCI was used for sample ionization, the vaporizer temperature was set to 450 °C, and the scan width was set to 1000 m/z with a scan time of 0.2 s and a MS acquire time of 10 min. Limonene in the dodecane phase was quantified by HPLC–MS/MS with a standard curve of limonene, with a linear response from 0.8 to 100 mg/L. Two biological replicates were used for each measurement and the data presented are the calculated average and standard deviation.

### Supplementary Information

Supplementary Material 1.

## Data Availability

All data generated during this study are included in this published article and supplementary information.

## Abbreviations

MVA: Mevalonate
Acetyl-CoA: Acetyl coenzyme A
NADPH: Nicotinamide adenine dinucleotide phosphate
LS: Limonene synthase
