# Supplementary material for: Efficient synthesis of limonene production in Yarrowia lipolytica by combinatorial engineering strategies
Source: Biotechnol Biofuels Bioprod. 2024 Jul 3;17:94. doi: 10.1186/s13068-024-02535-z (PMC11223395; doi:10.1186/s13068-024-02535-z)
Supplement: Supplementary file 1 — Supplementary Material 1. [file 13068_2024_2535_MOESM1_ESM.docx]

**Supplementary Information**

Efficient synthesis of limonene production in *Yarrowia lipolytica* by combinatorial engineering strategies

Young-Kyoung Park^1,2^, Lara Sellés Vidal^1^, David Bell^3^, Jure Zabret^4^, Mladen Soldat^4^, Martin Kavšček^4^, Rodrigo Ledesma-Amaro^1,*^

Correspondence: Rodrigo Ledesma-Amaro ([r.ledesma-amaro@imperial.ac.uk](mailto:r.ledesma-amaro@imperial.ac.uk))

1. Department of Bioengineering and Centre for Synthetic Biology, Imperial College London, London SW72AZ, UK

2. Université Paris-Saclay, INRAE, AgroParisTech, Micalis Institute, 78350 Jouy-en-Josas, France

3. SynbiCITE Innovation and Knowledge Centre, Imperial College London, London SW7 2AZ, U.K.

4. Acies Bio d.o.o., Tehnološki Park 21, SI-1000 Ljubljana, Slovenia

Supplementary Table 1. Protein sequences of genes used in this study.

| Gene name | Sequence |
| --- | --- |
| (D)-LS from *Citrus limon* | MDRRSANYQPSIWDHDFLQSLNSNYTDETYRRRAEELKGKVKIAIKDVTEPLDQLELIDNLQRLGLAYRFETEIRNILHNIYNNNKDYVWRKENLYATSLEFRLLRQHGYPVSQEVFNGFKDDQGGFIFDDFKGILSLHEASYYSLEGESIMEEAWQFTSKHLKEVMISKSMEEDVFVAEQAKRALELPLHWKVPMLEARWFIHVYEKREDKNHLLLELAKMEFNTLQAIYQEELKEISGWWKDTGLGEKLSFARNRLVASFLWSMGIAFEPQFAYCRRVLTISIALITVIDDIYDVYGTLDELEIFTDAVARWDINYALKHLPGYMKMCFLALYNFVNEFAYYVLKQQDFDMLLSIKNAWLGLIQAYLVEAKWYHSKYTPKLEEYLENGLVSITGPLIIAISYLSGTNPIIKKELEFLESNPDIVHWSSKIFRLQDDLGTSSDEIQRGDVPKSIQCYMHETGASEEVAREHIKDMMRQMWKKVNAYTADKDSPLTRTTTEFLLNLVRMSHFMYLHGDGHGVQNQETIDVGFTLLFQPIPLEDKDMAFTASPGTKG* |
| (L)-LS from *Mentha spicata* | MERRSGNYNPSRWDVNFIQSLLSDYKEDKHVIRASELVTLVKMELEKETDQIRQLELIDDLQRMGLSDHFQNEFKEILSSIYLDHHYYKNPFPKEERDLYSTSLAFRLLREHGFQVAQEVFDSFKNEEGEFKESLSDDTRGLLQLYEASFLLTEGETTLESAREFATKFLEEKVNEGGVDGDLLTRIAYSLDIPLHWRIKRPNAPVWIEWYRKRPDMNPVVLELAILDLNIVQAQFQEELKESFRWWRNTGFVEKLPFARDRLVECYFWNTGIIEPRQHASARIMMGKVNALITVIDDIYDVYGTLEELEQFTDLIRRWDINSIDQLPDYMQLCFLALNNFVDDTSYDVMKEKGVNVIPYLRQSWVDLADKYMVEARWFYGGHKPSLEEYLENSWQSISGPCMLTHIFFRVTDSFTKETVDSLYKYHDLVRWSSFVLRLADDLGTSVEEVSRGDVPKSLQCYMSDYNASEAEARKHVKWLIAEVWKKMNAERVSKDSPFGKDFIGCAVDLGRMAQLMYHNGDGHGTQHPIIHQQMTRTLFEPFA* |
| tHMGR | MTQSVKVVEKHVPIVIEKPSEKEEDTSSEDSIELTVGKQPKPVTETRSLDDLEAIMKAGKTKLLEDHEVVKLSLEGKLPLYALEKQLGDNTRAVGIRRSIISQQSNTKTLETSKLPYLHYDYDRVFGACCENVIGYMPLPVGVAGPMNIDGKNYHIPMATTEGCLVASTMRGCKAINAGGGVTTVLTQDGMTRGPCVSFPSLKRAGAAKIWLDSEEGLKSMRKAFNSTSRFARLQSLHSTLAGNLLFIRFRTTTGDAMGMNMISKGVEHSLAVMVKEYGFPDMDIVSVSGNYCTDKKPAAINWIEGRGKSVVAEATIPAHIVKSVLKSEVDALVELNISKNLIGSAMAGSVGGFNAHAANLVTAIYLATGQDPAQNVESSNCITLMSNVDGNLLISVSMPSIEVGTIGGGTILEPQGAMLEMLGVRGPHIETPGANAQQLARIIASGVLAAELSLCSALAAGHLVQSHMTHNRSQAPTPAKQSQADLQRLQNGSNICIRS* |
| ERG20m  (red: mutation) | MSKAKFESVFPRISEELVQLLRDEGLPQDAVQWFSDSLQYNCVGGKLNRGLSVVDTYQLLTGKKELDDEEYYRLALLGWLIELLQAFWLVSDDIMDESKTRRGQPCWYLKPKVGMIAIWDAFMLESGIYILLKKHFRQEKYYIDLVELFHDISFKTELGQLVDLLTAPEDEVDLNRFSLDKHSFIVRYKTAYYSFYLPVVLAMYVAGITNPKDLQQAMDVLIPLGEYFQVQDDYLDNFGDPEFIGKIGTDIQDNKCSWLVNKALQKATPEQRQILEDNYGVKDKSKELVIKKLYDDMKIEQDYLDYEEEVVGDIKKKIEQVDESRGFKKEVLNAFLAKIYKRQK* |
| NDPS1 from *Solanum lycopersicum* | MSARGLNKISCSLNLQTEKLCYEDNDNDLDEELMPKHIALIMDGNRRWAKDKGLEVYEGHKHIIPKLKEICDISSKLGIQIITAFAFSTENWKRSKEEVDFLLQMFEEIYDEFSRSGVRVSIIGCKSDLPMTLQKCIALTEETTKGNKGLHLVIALNYGGYYDILQATKSIVNKAMNGLLDVEDINKNLFDQELESKCPNPDLLIRTGGEQRVSNFLLWQLAYTEFYFTNTLFPDFGEEDLKEAIMNFQQRHRRFGGHTY* |
| ERG20m/(D)-LS (blue: linker) | MSKAKFESVFPRISEELVQLLRDEGLPQDAVQWFSDSLQYNCVGGKLNRGLSVVDTYQLLTGKKELDDEEYYRLALLGWLIELLQAFWLVSDDIMDESKTRRGQPCWYLKPKVGMIAIWDAFMLESGIYILLKKHFRQEKYYIDLVELFHDISFKTELGQLVDLLTAPEDEVDLNRFSLDKHSFIVRYKTAYYSFYLPVVLAMYVAGITNPKDLQQAMDVLIPLGEYFQVQDDYLDNFGDPEFIGKIGTDIQDNKCSWLVNKALQKATPEQRQILEDNYGVKDKSKELVIKKLYDDMKIEQDYLDYEEEVVGDIKKKIEQVDESRGFKKEVLNAFLAKIYKRQKGSGSGSGSGSDRRSANYQPSIWDHDFLQSLNSNYTDETYRRRAEELKGKVKIAIKDVTEPLDQLELIDNLQRLGLAYRFETEIRNILHNIYNNNKDYVWRKENLYATSLEFRLLRQHGYPVSQEVFNGFKDDQGGFIFDDFKGILSLHEASYYSLEGESIMEEAWQFTSKHLKEVMISKSMEEDVFVAEQAKRALELPLHWKVPMLEARWFIHVYEKREDKNHLLLELAKMEFNTLQAIYQEELKEISGWWKDTGLGEKLSFARNRLVASFLWSMGIAFEPQFAYCRRVLTISIALITVIDDIYDVYGTLDELEIFTDAVARWDINYALKHLPGYMKMCFLALYNFVNEFAYYVLKQQDFDMLLSIKNAWLGLIQAYLVEAKWYHSKYTPKLEEYLENGLVSITGPLIIAISYLSGTNPIIKKELEFLESNPDIVHWSSKIFRLQDDLGTSSDEIQRGDVPKSIQCYMHETGASEEVAREHIKDMMRQMWKKVNAYTADKDSPLTRTTTEFLLNLVRMSHFMYLHGDGHGVQNQETIDVGFTLLFQPIPLEDKDMAFTASPGTKG* |
| ERG20m/(L)-LS (blue: linker) | MSKAKFESVFPRISEELVQLLRDEGLPQDAVQWFSDSLQYNCVGGKLNRGLSVVDTYQLLTGKKELDDEEYYRLALLGWLIELLQAFWLVSDDIMDESKTRRGQPCWYLKPKVGMIAIWDAFMLESGIYILLKKHFRQEKYYIDLVELFHDISFKTELGQLVDLLTAPEDEVDLNRFSLDKHSFIVRYKTAYYSFYLPVVLAMYVAGITNPKDLQQAMDVLIPLGEYFQVQDDYLDNFGDPEFIGKIGTDIQDNKCSWLVNKALQKATPEQRQILEDNYGVKDKSKELVIKKLYDDMKIEQDYLDYEEEVVGDIKKKIEQVDESRGFKKEVLNAFLAKIYKRQKGSGSGSGSGSERRSGNYNPSRWDVNFIQSLLSDYKEDKHVIRASELVTLVKMELEKETDQIRQLELIDDLQRMGLSDHFQNEFKEILSSIYLDHHYYKNPFPKEERDLYSTSLAFRLLREHGFQVAQEVFDSFKNEEGEFKESLSDDTRGLLQLYEASFLLTEGETTLESAREFATKFLEEKVNEGGVDGDLLTRIAYSLDIPLHWRIKRPNAPVWIEWYRKRPDMNPVVLELAILDLNIVQAQFQEELKESFRWWRNTGFVEKLPFARDRLVECYFWNTGIIEPRQHASARIMMGKVNALITVIDDIYDVYGTLEELEQFTDLIRRWDINSIDQLPDYMQLCFLALNNFVDDTSYDVMKEKGVNVIPYLRQSWVDLADKYMVEARWFYGGHKPSLEEYLENSWQSISGPCMLTHIFFRVTDSFTKETVDSLYKYHDLVRWSSFVLRLADDLGTSVEEVSRGDVPKSLQCYMSDYNASEAEARKHVKWLIAEVWKKMNAERVSKDSPFGKDFIGCAVDLGRMAQLMYHNGDGHGTQHPIIHQQMTRTLFEPFA* |

Supplementary Table 2. Primers used in this study.

| Name | Sequence (5' - 3') |
| --- | --- |
| pTEF-200-Fw | CCATGCCGGACGCAAAATAGACTAC |
| TLIP2-Rev | CGATTTGTCTTAGAGGAACGCATATACAGTAATC |
| GGP-(D) LS-Rev | CTGACGTCTCAGGTCGGTCTCATAGATTAGCCCTTGGTGCC |
| (D) LS-800-Rev | CGTCGGCAGTAGGCGAACTG |
| (D) LS-Pero1-Fw | GCATCGTCTCATCGGGGTCTCAAATGGACCGACGATCTGCCAAC |
| (D) LS-Pero1-Rev | CTGACGTCTCAGGTCGGTCTCATAGATTAAAGCTTGGACTTGAACTGGTCCTCAGTGACACCGGCACCCATGCCCTTGGTGCCGGGAGAAG |
| (D)LS-pero2-Rev | CTGACGTCTCAGGTCGGTCTCATAGATTATAGTTTAGAAGAACCACCACCGCCCTTGGTGCCGGGAG |
| (L) LS-500-Rev | GGCAGACTCGAGGGTAGTCTCG |
| (L) LS-800-Fw | GCTCGAATCATGATGGG |
| (L) LS-Rev | CTGACGTCTCAGGTCGGTCTCATAGATTAGGCGAAGGGCTC |
| tHMG-500-Rev | CCAGCGTTGATGGCCTTGCAG |
| tHMG-800-Rev | CGCCCTTAGAGATCATGTTCATGCC |
| tHMG-pero-Fw | GCATCGTCTCATCGGGGTCTCAAATGACCCAGTCTGTGAAGGTGGTC |
| tHMG-pero-Rev | CTGACGTCTCAGGTCGGTCTCATAGATTATAGTTTAGAAGAACCACCACCAGATCGGATGCAGATGTTAGAGCCG |
| NDPI-ORF-Fw | ATGTCTGCCCGAGGCCTGAAC |
| NDPI-700-Rev | CGGTGTCGCTGCTGGAAGTTC |
| IDI1-Fw | GCATCGTCTCATCGGGGTCTCAAATGACGACGTCTTACAGCGACAAAATC |
| IDI1-550-Rev | CGGGTGAGGAAATGGAACTTGTC |
| IDI1-Rev | CTGACGTCTCAGGTCGGTCTCATAGACTACTTGATCCACCGCCGAATCTC |
| IDI-pero-Rev | CTGACGTCTCAGGTCGGTCTCATAGACTATAGTTTAGAAGAACCACCACCCTTGATCCACCGCCGAATCTCG |
| ERG8-pero-Fw | GCATCGTCTCATCGGGGTCTCAAATGACCACCTATTCGGCTCCG |
| ERG8-800-Rev | GATGGTTCCAGAGTCACCTTCCACTTTTG |
| ERG8-900-Rev | GCTTGCGCAGGTCGTTGAAC |
| ERG8-pero-Rev | CTGACGTCTCAGGTCGGTCTCATAGACTATAGTTTAGAAGAACCACCACCCTTGAACCCCTTCTCGAGCCG |
| ERG10-pero-Fw | GCATCGTCTCATCGGGGTCTCAAATGCGACTCACTCTGCCCC |
| ERG10-700-Rev | ACAGGCTTGAGGCCCTTGAGC |
| ERG10-pero-Rev | CTGACGTCTCAGGTCGGTCTCATAGACTATAGTTTAGAAGAACCACCACCCTCGACAGAAGACACCTTCTTGATGAC |
| ERG12-pero-Fw | GCATCGTCTCATCGGGGTCTCAAATGGACTACATCATTTCGGCGC |
| ERG12-1000-Rev | GCTGCATTTCCGAACCGATATCTC |
| ERG12-pero-Rev | CTGACGTCTCAGGTCGGTCTCATAGACTATAGTTTAGAAGAACCACCACCATGGGTCCAGGGACCGATG |
| ERG12-Rev | TGCAGTGGTCTCCTCGGTCTCATAGACTAATGGG |
| ERG12-Fw | GCATCGTCTCATCGGTCTCAAATGGACTACATCATTTCGGCG |
| ERG12-1000-Rev | GCTGCATTTCCGAACCGATATCTCTC |
| GGP-ERG12-Rev | CCGCGTCTCAGGTCGGTCTCATAGACTAATGGGTCCAGGGACCG |
| ERG13-pero-Fw | GCATCGTCTCATCGGGGTCTCAAATGTCGCAACCCCAGAACGTTG |
| ERG13-600-Rev | GGCTTGTAGAAATCGTAGGCATGC |
| ERG13-pero-Rev | CTGACGTCTCAGGTCGGTCTCATAGACTATAGTTTAGAAGAACCACCACCCTGCTTGATCTCGTACTTTCGTCGG |
| ERG19-pero-Fw | GCATCGTCTCATCGGGGTCTCAAATGATCCACCAGGCCTCCAC |
| ERG19-500-Rev | CGTAGCCTCCGTAGAGAGATCGAC |
| ERG19-pero-Rev | CTGACGTCTCAGGTCGGTCTCATAGACTATAGTTTAGAAGAACCACCACCCTTGCTGTTCTTCAGAGAACCATCCTC |
| GGP-Erg20-Fw | GCATCGTCTCATCGGGGTCTCAAATGTCTAAGGCCAAGTTCG |
| Erg20m-ORF-Fw | ATGTCTAAGGCCAAGTTCGAGTCTGTG |
| Erg20m-500-Rev | CGGGAGCAGTCAGCAGGTC |
| ERG20m-900-Fw | CGACGACATGAAGATCGAGC |
| ERG20m-pero1-Fw | GCATCGTCTCATCGGGGTCTCAAATGTCTAAGGCCAAGTTCGAGTCTGTG |
| ERG20m-pero1-Rev | CTGACGTCTCAGGTCGGTCTCATAGATTAAAGCTTGGACTTGAACTGGTCCTCAGTGACACCGGCACCCATCTTCTGTCGCTTGTAGATCTTGGCC |
| ERG20m-pero2-Rev | CTGACGTCTCAGGTCGGTCTCATAGATTATAGTTTAGAAGAACCACCACCCTTCTGTCGCTTGTAGATCTTGGCCA |

Supplementary Table 3. Limonene production in *Y. lipolytica*.

| **Parent strain** | **Origin of limonene synthase** | **Genetic modification** | **Limonene production** | **Culture condition** | **Reference** |
| --- | --- | --- | --- | --- | --- |
| po1f | Agastache rugosa (D) | NDPS1,  HMG1, ERG12 | 23.56 mg/L | Flask | Cao et al. 2016 |
| po1f | Agastache rugosa (D) * 2 copies | NDPS1,  HMG1, ERG12 | 40.7 mg/L  165.3 mg/L | Flask  Fed-batch | Cheng et al. 2019 |
| po1g | Citrus limon (D) | HMG1 | 11.705 mg/L | Fed-batch | Pang et al. 2019 |
| po1g | Mentha spicata (L) | HMG1 | 11.088 mg/L | Fed-batch | Pang et al. 2019 |
| po1f | Agastache rugosa (D) * 2 copies | NDPS1,  HMG1, ERG12 | 20.57 mg/L | Flask | Yao et al. 2020 |
| ATCC20460 | Perilla frutescens | HMG1, ERG12, ACL1, SeACS, IDI, ERG20m, HMG, Lowering SQS |  | Glass tube | Arnesen et al. 2020 |
| po1g | Citrus limon (D) | HMG1, NDPS1, IDI | 37.70 mg/L  91.24 mg/L | Flask  Fed-batch | Li et al. 2022 |
| po1g | Mentha spicata (L) | HMG1, NDPS1, IDI | 36.74 mg/L  83.06 mg/L | Flask  Fed-batch |  |
| po1d | Citrus limon (D) * 3 copies | Fusion of LS and ERG20  HMG1  Multi-copy | 24.8 mg/L | Flask | This study |
| po1d | Mentha spicata (L) * 3 copies | Fusion of LS and ERG20  HMG1  Multi-copy | 29.4 mg/L | Flask | This study |
| po1d | Citrus limon (D) in peroxisome | MVA pathway in peroxisome | 47.8 mg/L | Flask | This study |
| po1d | Citrus limon (D) in peroxisome | MVA pathway in peroxisome | 69.3 mg/L | Fed-batch | This study |
